# What is the 'problem' that outreach work seeks to address and how might it be tackled? Seeking theory in a primary health prevention programme

**DOI:** 10.1186/1472-6963-11-350

**Published:** 2011-12-28

**Authors:** Mhairi Mackenzie, Fiona Turner, Stephen Platt, Maggie Reid, Yingying Wang, Julia Clark, Sanjeev Sridharan, Catherine A O'Donnell

**Affiliations:** 1 Urban Studies, School of Social & Political Sciences/Institute of Health &Wellbeing, University of Glasgow, 27 Bute Gdns, Glasgow, UK, G12 8RS; 2 General Practice & Primary Care/Institute of Health & Wellbeing, University of Glasgow, 1 Horslethill Rd, Glasgow, UK, G12 9LX; 3 Centre for Population Health Sciences, Medical School, University of Edinburgh, Teviot Place, Edinburgh, UK, EH8 9AG; 4 MRC Social & Public Health Sciences Unit, 4 Lilybank Gardens, Glasgow, UK, G12 8RZ; 5 Institute of Applied Health Research, 70 Cowcaddens Rd, Glasgow Caledonian University, Glasgow, UK, G4 OBA; 6 St. Michael's Hospital, University of Toronto, 30 Bond Street, Toronto, Ontario, Canada, M5B 1W8

## Abstract

**Background:**

Preventive approaches to health are disproportionately accessed by the more affluent and recent health improvement policy advocates the use of targeted preventive primary care to reduce risk factors in poorer individuals and communities. Outreach has become part of the health service response. Outreach has a long history of engaging those who do not otherwise access services. It has, however, been described as eclectic in its purpose, clientele and mode of practice; its effectiveness is unproven.

Using a primary prevention programme in the UK as a case, this paper addresses two research questions: what are the perceived problems of non-engagement that outreach aims to address; and, what specific mechanisms of outreach are hypothesised to tackle these.

**Methods:**

Drawing on a wider programme evaluation, the study undertook qualitative interviews with strategically selected health-care professionals. The analysis was thematically guided by the concept of 'candidacy' which theorises the dynamic process through which services and individuals negotiate appropriate service use.

**Results:**

The study identified seven types of engagement 'problem' and corresponding solutions. These 'problems' lie on a continuum of complexity in terms of the challenges they present to primary care. Reasons for non-engagement are congruent with the concept of 'candidacy' but point to ways in which it can be expanded.

**Conclusions:**

The paper draws conclusions about the role of outreach in contributing to the implementation of inequalities focused primary prevention and identifies further research needed in the theoretical development of both outreach as an approach and candidacy as a conceptual framework.

## Background

Across the statutory and voluntary sectors outreach work has a long history as a means of engaging individuals and communities, typically those marginalised by processes of social exclusion and socioeconomic deprivation [1]. Within the health domain, since the outbreak of the HIV and AIDS epidemic of the 1980s, outreach activities have focused on harm reduction in relation to sexual health and substance abuse, as well as in the field of mental health [1,2]. Whilst outreach, as embodied by home visits, has historically had a place in primary care through the work of health visitors, district nurses and general practitioners, outreach more generally has not traditionally had a significant role in the delivery of primary care prevention programmes.

Nonetheless, it is now widely accepted that preventive approaches to health are disproportionately accessed by the more affluent in society, thus increasing (rather than reducing) inequalities in health outcomes between different social strata [3-6]. As a result, recent international health improvement policies have advocated the use of targeted, preventive primary care as a means of reducing risk factors in poorer individuals and communities [7-9]. In the UK, outreach work has become one part of primary care's response to this challenge within a broader set of reach and engagement strategies [10-12].

However, outreach has been described as eclectic in its purpose, client group and specific mode of practice and, as a direct result of this heterogeneity, little is known about its effectiveness. Ottoson and Green have suggested that the concept of outreach needs to be more carefully considered, with typologies of different approaches and their underlying mechanisms delineated prior to assessment of their effectiveness [13]. In other words, outreach approaches need to be better theorised. Such a recommendation fits with a growing consensus within the evaluation community that impact assessment without adequate theory does not constitute good science [14-16].

Keep Well was launched in 2006 as a national approach to tackling cardiovascular mortality and morbidity through primary care, targeting those in areas of severe socio-economic deprivation [17,18]. It was based on an anticipatory care approach [17,19] and, amongst other strategies, made use of outreach to engage its target population in health checks and subsequent interventions. Using Keep Well as a case, this paper addresses the question of what types of engagement 'problem' outreach aims to solve and identifies implicit and explicit 'theories' of how these problems might be addressed using outreach approaches.

### Background to outreach as an approach

According to several commentators 'outreach work' acts as an umbrella term covering a wide range of activities designed to bridge both physical and ideological gaps between users and services (for example [20]). The concept of users' 'non-engagement' with services is central to the discussion of outreach work and is used to signify an inability or unwillingness to engage with services that have been deemed appropriate for particular groups. This overarching 'problem' is, however, rarely unpacked to make transparent the more specific issues that constitute, or lead to, a lack of fit between users and services. Furthermore, the particular groups that outreach aims to engage have historically been identified within 'classic' areas of harm-reduction (sexual health, HIV risk-reduction, substance abuse and mental health). This raises questions about the types of problems that outreach located within primary care, and more specifically in relation to anticipatory care within the broader field of tackling health inequalities, aims to address.

More emphasis has been placed on describing models of outreach activity than on understanding the theorised links between problems and the mechanisms by which outreach aims to address these problems [13]. Across the proliferation of outreach projects, models operate on a continuum of engagement and vary in the extent to which they aim to address problems at an individual or structural level. Authors voice concerns, therefore, that services continue to implement outreach activity without clarifying what types of outreach (with which specific mechanisms) are suited to generating positive outcomes in particular circumstances (for example [21]).

### Keep Well as a test bed for theorising the practice of outreach for primary prevention

Keep Well was a multi-million pound investment in primary care that emerged from policy documents advocating a key role for the Scottish NHS in reducing health inequalities [18]. Its aim was to reduce inequalities in cardiovascular morbidity and mortality through improved reach of primary prevention programmes. Initially targeted at five pilots in the most deprived areas of Scotland, it provided incentives for general practices within those pilot sites to develop reach and engagement strategies to encourage registered patients between the ages of 45 and 64 to attend for a health check and to sustain their involvement in subsequent treatment. Treatment included pharmacological interventions, lifestyle advice, motivational interviewing and referrals to wider social and material supports. The programme (as with prevention approaches more generally) was predicated on the view that, for those whose modifiable risk factors are identified early, there are evidence-based interventions that can be used to reduce the risk of clinical events. The evidence base for their role in tackling inequalities is not, however, strong.

Keep Well was based, therefore, on a model of anticipating health needs before they become manifest (thereby challenging a reactive, episodic model of care provision) and delivering continuous, integrated, preventive care with the patient as partner [19,22]. Such a model of care has been termed anticipatory care and has its roots in the work of Van den Dool in the Netherlands and Tudor Hart in Wales [23,24].

The challenge that the programme aimed to meet was that of engaging with patient and population groups who are traditionally less likely to be in contact with mainstream services [25]. Across a range of health conditions those living in more deprived circumstances have been found to make less use of preventive health services than their more affluent counterparts [26].

The kinds of explanations that are given for this socially-patterned disparity relate to both demand and supply. In terms of factors that may help to shape demand for preventive health care, there is evidence that those in more deprived groups are less likely to act on early signs of ill-health because of the normalisation of poor health within poorer communities [27] and because of a concern that they will be held responsible for their illness [28].

On the supply side there is a long-standing literature, for example, that demonstrates the under-resourcing of primary care in relation to need [29] and that posits that under-resourcing stems from a lack of political and managerial understanding of the disproportionate resources required to deliver a level-playing field in relation to care never mind an augmented service [30].

A construct developed by Dixon-Woods and colleagues [4,5] provides a helpful explanatory bridge between explanations that focus on expressed patient needs and health service responses. They argue that 'candidacy' [The term candidacy as utilised by Dixon Woods and colleagues is broaden than that used in other medical sociology/anthropology writings. For example, as used by Davison, Davey-Smith & Frankel [31] it refers more narrowly to lay views of what constitutes a typical individual with a heart problem.] for having a particular medical condition or for receiving health services is socially constructed and influenced by one's social circumstances, cultural norms and interaction with health care professionals and systems. It is argued that people living in more disadvantaged circumstances tend to normalise symptoms of ill-health because of a higher prevalence of such symptoms, and tend to manage their health as a series of minor and major crises rather than by prevention and positive maintenance [4]. The construct of candidacy, therefore, attempts to explain the complex vulnerabilities, contexts and processes that influence how people, and healthcare systems, come to define eligibility for healthcare [4]. Such eligibility is negotiated prior to initial contact with services and through subsequent journeys through the health service. They describe this dynamic set of factors influencing access to care as follows [4]:

*"We suggest that social patterning of perceptions of health and health services, difficulties in marshalling the practical and social resources needed to use services, a lack of alignment between the priorities of disadvantaged people and the organisational values of health services, conspire to create vulnerabilities for socially disadvantaged people in their negotiation of health services" (p. 98)*.

In reactive help-seeking behaviour, people present themselves to the healthcare system once they have recognised and acted on their candidacy; however, in the case of preventive health, this help-seeking is reversed in that it is the healthcare system that has identified certain features of candidacy, which are then negotiated with those individuals who fall within particular target groups [4]. In this case recipients of invitations are asked to recognise and accept the candidacy that is presented to them.

For anticipatory care initiatives such as Keep Well, important questions therefore arise as to whether those targeted by the intervention recognise their candidacy, firstly in relation to preventive health in general, and, secondly, in relation to the specific health issue(s) that the initiative hopes to address. In respect of Keep Well, the issue is whether people see themselves as candidates for cardiovascular disease and/or for preventive treatments to reduce the risk of such disease. The key question is: to what extent is their perception of their own candidacy for *disease *and *its prevention *in alignment with those of the pilots and practices that have already defined their eligibility? Candidacy also looks, however, at how the health service can influence notions of eligibility, including a focus on access, capacity, flexibility of services as well as on the many one-to-one interactions between patients and professionals. Barriers erected by systems and individual professionals are therefore seen to be important factors that overlap with, and influence, perceptions of candidacy.

Keep Well's approach to stimulating candidacy was flexible but, at the same time, remarkably conservative and, in the main, not targeted at social and material determinants of non-engagement. Individual pilots/practices were given freedom in relation to how they contacted their target population and were able to use a range of methods introduced at different stages [32].

The 'problems' that outreach work in Keep Well hoped to address, however, were less explicit than the overarching notion of the need for a novel approach to reaching and engaging hard-to-reach groups. This study aims to delineate the theories (both implicit and explicit) of professionals responsible for planning and implementing outreach approaches within Keep Well. It asks: what is the 'problem' that outreach work seeks to address and how is it tackled'?

## Methods

The study reported in this paper was part of the national multi-method evaluation of Keep Well (a full description of the methods used across the evaluation is reported elsewhere [33]).

In an early, formative phase of the evaluation outreach had emerged as a newly developing means of addressing the challenge of reaching a sizeable group of individuals who were not responding to postal and telephone invitations to attend a health check [34]. In the context of Keep Well, therefore, outreach was viewed at national and local levels as 'innovative'; it was, therefore, selected as a standalone study for further exploration. Ethical approval was granted for the study in 2007 by the University of Glasgow Medical Faculty Ethics Committee; it required that informed consent be gathered from all participants and that the data be presented in an anonymised format.

The outreach study was undertaken in four locations across Scotland. Within these, semi-structured interviews were conducted with all staff directly involved in outreach work as part of the Keep Well programme (N = 21). This included those in explicitly defined outreach posts (entitled 'outreach workers') and those identified as carrying out outreach approaches as part of a wider role. To put the work of these staff within a strategic context, interviews were also held with their line-managers and with the Keep Well project managers.

Key thematic questions covered within the semi-structured interviews were: the purpose of outreach activity; the roles and procedures undertaken by professionals; professionals' views of why particular individuals needed outreach; and the perceived impact of the approach. Data collection was deemed to be complete when all individuals identified for participation had been interviewed.

Interviews were transcribed and the data organised into thematic categories as above using *Framework*, an approach with associated software developed by the National Centre for Social Research specifically for qualitative policy evaluation [35]. Framework Analysis involves the following five key stages: familiarisation through re-reading of the transcripts; development of a thematic framework; indexing of the transcripts using the framework; charting of each transcript by extracting coded data into a series of thematic charts; and mapping and interpretation by comparing thematic charts across interviewees.

A thematic (coding) framework was identified based on both *a priori *themes and emergent themes from the familiarisation stage. The final stage of analysis was data mapping and interpretation in relation to the predefined categories and emerging themes, and, explicit attempts to identify alternative interpretations were made. Emergent themes focused on differing conceptualization of the 'problems' of non-engagement; data analysis workshops involving the research team were conducted throughout the evaluation and used to critically appraise emerging analyses. In the following presentation of findings each illustrative quotation is followed by the unique anonymised code of the participant. This consists of a letter which indicates which of four geographical areas the individual worked in and their participant number within each site.

### Limitations of the study

The main potential limitation of the study was that the researchers were part of a wider evaluation team of a high-profile national programme and, therefore, viewed as playing a role in decision-making about sustained national and local funding for anticipatory care and the Keep Well approach. This may have had an impact on the extent to which participants were prepared to reflect on perceived weaknesses or problems within the approach. Similarly, practitioners may have felt obliged to participate in a study directly associated with their employment. There was, however, no evidence in the data nor in the manner of participants that would directly support either of these potential problems. In partial mitigation we gave assurance that key workers could withhold consent to participate without being named and where individuals might be identified by their role or where their views were deemed negative in relation to Keep Well they were given the opportunity to view direct quotations that we planned to utilise for reporting purposes and to request their removal. No such requests were made.

## Results

### The purpose of outreach within Keep Well

Across the four locations outreach was developed in response to the limitations of earlier attempts within the Keep Well programme to reach and engage the target population. These attempts were, largely, relatively traditional, with an initial focus on sending letters with fixed or open appointment times to targeted individuals. Practices found this method useful in engaging part of their population but not all of it. This is consistent with the findings of a review of the access literature [4] which found that appointment systems requiring people to attend a particular place at a specified time are likely to incur high levels of non-engagement amongst the most deprived. In Keep Well telephone approaches were also used to encourage attendance amongst those who had not responded to letters and patients were 'opportunistically' invited as they attended the surgery for another reason; outreach became a common feature across the programme as significant parts of the target population remained unengaged [34].

**Figure 1:**
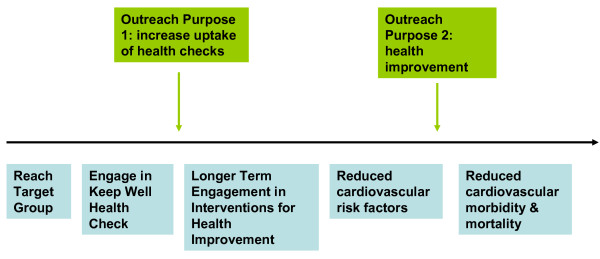
**The Purposes of Outreach as part of the overall Keep Well approach**.

Despite the common driver of improving on these approaches, the original purpose of outreach varied between pilots. In two areas (A & B) the initial purpose was to increase the number of individuals attending for an initial health check; in the remaining two areas (C & D) outreach initially focused on the longer term engagement of individuals in health improvement interventions. Figure 1 - 'The Purposes of Outreach as part of the overall Keep Well approach' illustrates how these two purposes fit with the logic of the wider Keep Well programme with its short-term goals of health service engagement and longer term health improvement and inequality reduction. However, these initially distinct approaches blurred over time as pilots learned from each other and changed their ways of working accordingly [36]. By the time that the case-study was conducted, all pilots described their work as having these two aims.

Aims expressed in this way, however, reveal little about tacit hypotheses relating to who are perceived to be hard to engage in preventive services and why; nor about the mechanisms by which outreach interventions might be anticipated to meet their goals; nor, indeed, what the goals of practitioners might be.

### The nature of outreach interventions in Keep Well

**Figure 2:**
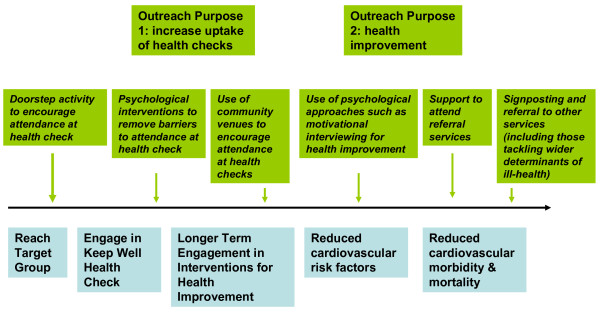
**Emerging outreach approaches**.

Six broad categories of intervention by those with an outreach remit were identified across the four wave 1 pilots. Figure 2 - 'Emerging Outreach Approaches' illustrates the alignement of these intervention types with the two primary purposes of outreach described above. Thus, in order to increase levels of attendance at the health check three main approaches were utilised: (1) 'doorstepping' people's homes to invite their participation or to provide health checks in situ; (2) providing support to remove psychological barriers to attendance (for example, motivational interviewing or solution-focused therapy); and (3) inviting participation of individuals encountered in community venues. To increase levels of engagement in health improvement three approaches were also taken: (1) the use of psychological approaches to facilitate lifestyle changes; (2) support to attend referrals to services within and outside the primary care practice; and (3) signposting and referral to other services (including those aimed at tackling social and structural determinants of poor health, such as welfare advice).

In describing their roles within these activities participants provided evidence, both implicitly and explicitly, of their theories about why engagement with primary prevention services was a problem for particular sub-groups of the population and mechanisms for generating positive outcomes. In the next section we explore these hypotheses and, where relevant, assess their fit with the concept of candidacy.

### Seven 'problems' and their outreach 'solutions'

**Figure 3:**
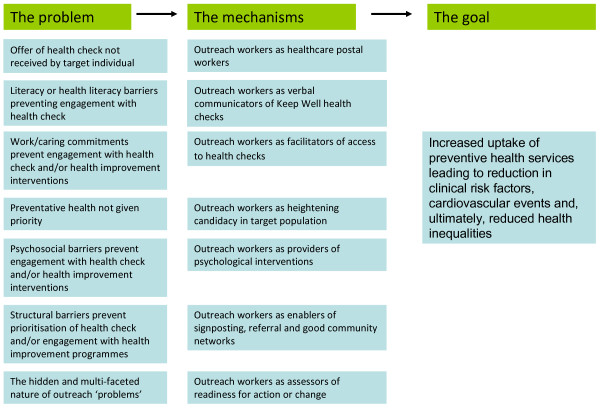
**Implicit theories of outreach within Keep Well**.

As outlined above, across the two purposes of outreach within Keep Well different types of outreach work were operating to solve seven non-engagement problems derived from the interview data; this mirrors the multi-faceted nature of outreach discussed earlier [13]. Figure 3 - Implicit theories of outreach within Keep Well summarises the pathways identified from problem through mechanisms to aim of intervention. Each of these is now described in turn.

#### Problem 1: target population do not receive their Keep Well Health Check invitation

Across the wave 1 pilots Keep Well experienced difficulties in reaching a group of patients within their target population because their contact details were incorrect. In this scenario, the message about an individual's candidacy for preventive services literally did not reach them. The limitations of existing practice registers were exacerbated in areas that were undergoing housing regeneration. In these circumstances outreach workers were therefore found to be working in the capacity of a **healthcare postal worker**, where health check invitations were personally delivered and practice registers updated to remove non-existent addresses and identify those who had moved on. As a result, there was a view that, over time, outreach work may provide a more realistic picture of those in 'hard-to-reach' categories by eliminating 'ghost' patients. Without this approach, the target population still to be reached and engaged would have been inflated. This problem is illustrated as follows:

"[t]here's certain barriers that we face with some of the information that we get from practices and it could be that people have moved away so you're going to empty buildings and stuff like that, or they haven't updated the practice that they have moved on to, so that kind of thing. They're hard to reach in terms of that, but in terms of going out and visiting them, they're not hard to reach."(A13)

Except where patients deliberately avoid their general practice (or avoid providing their practice with up-to-date contact details), this 'problem' could be tackled by more proactive practice efforts to update their patient registers and does not require intervention by skilled health professionals.

#### Problem 2: literacy or health literacy barriers preventing engagement with health checks

Given the demographics of the areas within which Keep Well was located, there was an expectation that part of the target population would have difficulties with literacy, e.g. reading letters of invitation. If telephone contact had also not succeeded then this problem could be overcome by visits from outreach workers to individuals in their homes. In addition, outreach workers reported instances where individuals were functionally literate but had not attended for a health check either because they believed that they had already had a health check (e.g. by virtue of attendance at a previous unrelated screening appointment), or because they did not understand the purpose of the health check or its place within the health system. One worker, for example, said:

"I think people need... rather than a letter going to their door and... even though we would put one of the blue Keep Well leaflets in to explain it, but it's not going into the detail that we can go into when we're at the door talking to them; it's a far better way of engaging with people rather than just sending a letter because if they've got any questions then they can just ask us right there and then we can tell them, so it is far better, definitely." (B21)

In these circumstances outreach workers can be argued to **bridge gaps in understanding **about preventive services and so contribute to solving the problem of health literacy, as described by Nutbeam [37].

#### Problem 3: work/caring commitments prevent engagement with the health check and/or subsequent health improvement interventions

Early in its implementation Keep Well had identified that one driver of non-attendance at health check appointments was the timing of appointments. In particular, feedback from patients had raised awareness of the problems experienced by the working population in attending appointments during the working day - a problem more prevalent for those in low-waged employment with little flexibility to take time out of their working day without losing income. To tackle this, some practices had made efforts to offer more flexible out-of-hours appointment times. Outreach workers were faced with a parallel access problem for those who had significant caring responsibilities and were able to offer practical assistance by providing health checks within the home. In this respect outreach workers were acting as **facilitators of access to health checks**. One worker, for example described the following case:

*"I've been on the doorstep with a person who was a carer and [they] actually said they couldn't take forty minutes out of their day to come to the health centre, to get a bus, to do it, and to get a carer, to get someone else in to care for her son... So then we had the opportunity to say 'well, have you got forty minutes just now... because we could come in now'. That was done there and then and we identified issues when we were doing the assessment... it was like a complete sort of package of care... you weren't leaving it cold at the doorstep, you were following it through" (C2)*.

#### Problem 4: preventive health not given priority

The existing literature on inequities in access to preventive services supports the view that those living in deprived circumstances are less likely to prioritise health maintenance than those living in more affluent areas [4], borne out by the current study but it is important to note that, once outreach staff made contact with individuals, they found only a minority of individuals who expressly stated that they did not want to know whether or not they were at risk of illness. One worker said, for example *"[we] do get some people that say 'no, I'm not interested, I don't want to know if I'm ill. You'll get that an odd time." (A13)*. Thus, the experience of outreach staff in the Wave 1 pilots was that candidacy for preventive services or for cardiovascular disease was rarely rejected outright by individuals.

On the other hand, it was often reported that patients found it difficult to find time to prioritise preventive health care. This is supported by the work of Goddard and Smith [38] who found that barriers of time that would be surmounted during a health crisis hindered the prioritisation of preventive health. For example:

"I think a lot of people that I speak to will say to me, 'If I'm sick then I'll go to the doctors.' They're busy, they've got busy lives; sometimes they've got chaotic lives and this is just another thing to do that they don't have time to do." (A15)

Outreach workers viewed pressure of time as another barrier preventing those with caring responsibilities from initial engagement in Keep Well. By visiting the homes of those with caring roles outreach staff were able to prompt individuals to consider their eligibility for Keep Well. In this way outreach was viewed as a means of **highlighting candidacy **for a group of individuals who have to deal with the ill-health of their parents, children and spouses on a daily basis. This **translational role **was deemed necessary because, as one worker said, "*if you're caring for somebody... you can't think about yourself." (C5) *and chimes with the long-established literature on how caring for others can be a risk factor in relation to one's one health [39,40].

#### Problem 5: psychosocial barriers prevent engagement with the health check and/or subsequent health improvement interventions

For another group of patients outreach had to move beyond the role of convincing individuals of their eligibility for Keep Well, and of the benefits of primary prevention for long-term health, into **tackling psychosocial problems preventing engagement**. Such problems were seen as barriers to engagement with initial health checks and/or the health improvement interventions to which Keep Well referred patients following the identification of clinical risk. One outreach manager explained that:

"Our outreach workers are very skilled too at going out and signposting patients to other agencies, where the patient might not be ready to come into Keep Well initially for a health check, but need other services in there at that particular time." (B18)

Pilot areas differed in the extent to which the provision of different forms of psychological interventions (including motivational interviewing) was considered to be part of the outreach worker's role. In two pilot locations such interventions were provided in the home by the outreach worker, while in the remaining two the role of the outreach worker was to identify problems and signpost/refer to other services.

#### Problem 6: structural barriers prevent prioritisation of health check and/or engagement with subsequent health improvement programmes

Two types of structural problem were highlighted by outreach staff. First, there was the need to fill an existing deficit in partnership working between primary care and other services of relevance to health improvement. For example, one manager stated that *"[the] [role] of an outreach worker in [C] was to help to bridge between GP practice and all the other services; with NHS, Council, voluntary sector that are out there" (C1)*. The second type of structural problem arose when a focus on health improvement goals was impeded by material disadvantage. Outreach workers reported spending considerable time attempting to address issues such as poor housing, unemployment and debt, predominantly through **signposting, referral and the mobilisation of good community networks**. The lengthy quotation below illustrates the perception that Keep Well was originally focused on a narrow behaviour change model but that a broader understanding of structural determinants helped to contextualise non-engagement. This offers the perspective that taking control of one's candidacy is problematic and/or irrelevant when the rest of one's life is shaped by poverty and/or negative psychosocial factors and is consistent with outreach research in the field of homelessness [41].

"I think the thinking was quite medical initially. Even although I heartily agree with health behaviour inputs, it's absolutely vital that we'll do that, I still think... the role of all these other [structural] barriers was strongly underestimated because basically, I mean, what is point in giving up smoking if... the rest of your life is so absolutely infuriatingly problematic. You know, there are all these other barriers so, you know, if we can help people to rid these other barriers of debt.... I mean for most people the chief priority is not eating five pieces of fruit a day but if you get rid of loads of other things and you motivate people, and you, you know, people feel that they have some control and power over their health... the five pieces of fruit a day becomes logical and understandable and reasonable, and 'I'll try and do that' and it becomes pleasurable because it's part of a future if... because I think again hopelessness is something that, you know, if people are optimistic they're planning ahead, they're future focused and they've got hope. But if they're not they're... if they're living in the moment with a whole bunch of issues that are weighing down on them they're not future focused. They are... they probably lack hope."(C5)

Outreach workers have a limited capacity to 'solve' this underlying reason for non-engagement in primary prevention, beyond sign-posting and referral and operating as a source of intelligence to GP practices. Operating within people's homes was, however, perceived to have provided insights into living conditions that put their non-attendance at a health check into perspective. One worker said, for example:

" That was a real eye opener... you go to the home and they haven't got a cooker or haven't got any furniture, it's just floorboards... maybe a microwave, you know." (C4)

#### Problem 7: The hidden and multi-faceted nature of outreach 'problems'

The final 'problem' of non-engagement that was identified was the hidden nature of the reasons for not attending a Keep Well health check. In other words, prior to making contact with the target individuals it was not possible for outreach workers to second guess the nature of the problem that they would be required to solve. The contingent nature of their role is illustrated by one outreach worker as follows:

"Our daily job is that we're given lists from GP practices to go out and find people that's like, hard to reach in terms of maybe their chaotic lifestyles; it could be that they're working all the time and they can't get into practices, they're carers to housebound people, that kind of thing.... you come up against a number of issues when you're out in the fields; you find a lot of people, it might be that they specifically don't want to come in. There could be areas that they've got phobias of needles and they don't want to come to the doctors. They could be a carer, for like, a person in their family, and they're unable to get of the house; there's people that have got agoraphobia and panic attacks and they just don't get out at all, but they certainly would like to take part in the health checks so it's kind of trying to kind of work a way to get all these people into the practice." (A13)

Problems were not only hidden behind doors but, not surprisingly, many individuals experienced multiple psychosocial and structural problems. This is captured by the following description:

"[There] was a couple that I went to and the husband has lost his job and he's been in the building trade and was made redundant about six months ago and he was quite down and you know, at home, so he's down already but he's a home so he's eating all the time and he's put on weight which is making him feel worse about himself, do you know, so there's always other issues in there but I suppose the success comes from maybe being able to help people deal with what's going on in their lives that are... you know, ultimately it affects their cardiovascular risk score because, you know, the lack of activity, the poor diet, the weight gain." (D9)

Once again, this points to **multiple entry points to accepting candidacy **for health improvement programmes, in general, and anticipatory care, in particular. These various entry points may also be more salient to individuals at different times and the outreach worker's role becomes one of **assessing readiness for action or change**. The following quotation from an outreach worker demonstrates the ways in which routes to health improvement are negotiated by patient and professional.

"He [the patient] was drinking and I can't remember what actually led into it, alcohol wasn't what he wanted to change; he wanted to stop smoking. So although I thought he should be stopping drinking, he thought smoking was the thing he felt was affecting his health more. So I almost feel that if you can tackle one, you can always get to the next one, does that make sense?... I mean I've seen that quite a lot of people, you tackle their weight but they stop smoking six months later. They start to think about stopping smoking." (D10)

This also highlights a type of intervention that is long-term in nature rather than delivered in a single-dose on the door-step. In addition, engagement was emphasised as a process rather than an event:

"Just because they're engaged in a health check doesn't mean to say they're going to maintain their engagement. If they've been difficult to engage with in the first place, okay it can be seen as an achievement to get them in, but if there's points of referral there or other areas that could be more helpful for that person, it's common sense to kind of follow through on that." (B22)

This quotation emphasises that it is unhelpful for programmes such as Keep Well to consider engagement as a single point of contact as opposed to an ongoing negotiation between users and services. This helps to explain why the initial dichotomy of outreach purpose as outlined in Figures 1 and 2 broke down in practice and led to a broader understanding of mechanisms by which engagement might be encouraged (Figure 3).

## Discussion

The current study has shown that the 'problem' of non-engagement with preventive health care, as experienced by outreach workers, is multidimensional. Outreach work 'solutions' which have been developed to address the problem in Keep Well could be incorporated into health improvement programmes of this type. In this final section of the paper we discuss the findings under three headings: the implications of the findings for the provision of anticipatory care within primary care; the contribution of the study to the theoretical concept of candidacy; and, the scope for further theoretical and empirical investigation.

### Anticipatory care

In order to maximise the potential benefits of taking an anticipatory approach to preventing ill-health, those who are not in receipt of optimal care need to be identified, alerted to their eligibility for such services, and engaged in the receipt/negotiation of care [19]. Imprecise tools exist to enable the health service to undertake this work. Identifying those most in need of preventive care is difficult: whilst those in the most deprived areas are less likely to make use of preventive services, not all individuals living in such areas will personally be deprived, and, of those who are, not all will be disinclined to use preventive services [22,25]. When it comes to making contact with the target population to invite them to arrange a health check, more traditional approaches of sending letters and making phone calls are successful for a proportion of the target population; however, despite numerous attempts, part of the target population remains unengaged [42]. Outreach offers a means of improving engagement among those previously unengaged, however, it throws open new challenges for the management and practice of primary care services. As outlined earlier, the first of these is the lack of a robust evidence base for specific outreach activities (we return to this at the end of our discussion). The findings from our study point to two main additional challenges: the hidden nature of non-engagement; and the extent to which the health care system is able to address structural causes of non-engagement.

As a result of the hidden nature of non-engagement, primary care cannot specify in advance the types of health care solutions that will work to engage its target population at an individual level. This has implications for how workloads and skill mixes of outreach staff are best managed and resonates with other health service use of outreach as a means of health improvement [43] and, pertinently, to the hidden work of GPs as they engage with their patients in consultations [44].

The current study demonstrates not only that the precise problem of non-engagement cannot be 'read' in advance by practitioners but that many of the issues faced by outreach workers, in the homes of the target population, derive from the social and structural determinants of health inequalities whose solutions lie outside the traditional remit of clinical primary care. If outreach approaches are to be part of primary care's efforts to increase engagement of poor communities in preventive health care then more determined action is required at both a community and a wider systems level [34,45].

The notion of a continuum of complexity developed in the current study offers a means of categorising non-engagement problems and of connecting these to a broader understanding of how they relate to the inequitable distribution of poor health outcomes in deprived neighbourhoods. One such broader understanding is that provided by the literature on candidacy.

### Candidacy

The findings from the current study both support and expand the theoretical concept of candidacy from the perspective of the health care professional. Implicitly and explicitly, the work of professionals with an outreach role in Keep Well was aimed at increasing the likelihood of candidacy being enacted by those whom they were able to reach. Dixon-Woods and colleagues describe stages of candidacy [4]. These begin with individual recognition of candidacy and continue with the presentation of the patient to services and adjudication on the validity of the candidacy claim by the health care professional. However, in the case of Keep Well (as with other preventive programmes) it is the health service that presents to the individual rather than the other way round and outreach workers used a range of differing approaches to augment the likelihood of candidacy being accepted. These included: efforts to deliver and reinforce messages of benefits to the individual in engaging in preventive programmes; encouraging some changes to the system, such as opening hours and improved accuracy of practice registers; provision of psychosocial and practical interventions aimed at reducing barriers to acceptance of interventions; and recognising that other barriers to engaging with services would rely on wider material/system change.

The final stage in the candidacy journey, as described by Dixon-Woods and colleagues [4], is converting engagement with health care interventions into improved health outcomes. Based on the findings from the current study we would argue that this is the weakest component of the concept. First, it over-simplifies the necessary pathways between acceptance of candidacy and engagement with prescribed interventions since it may require (re)establishing candidacy at an individual level for a series of different 'treatments', including maintenance of a statin regimen, adoption and continued engagement with lifestyle change, acceptance of psychosocial interventions and seeking and receiving material assistance. This broad range of 'problems' requires action by public and third sector organisations beyond the health service. Consequently, the 'operating conditions' of all such organisations and the connections between them need to be understood in relation to the question of how individuals travel through the system of health care. Second, and perhaps more importantly, this final stage somewhat downplays the extent to which structural determinants of health conspire to undermine positive health changes by individuals. Thus, a key deterrent to voicing candidacy for cardiovascular disease and accepting preventive treatments is the perceived difficulty of sustaining the likely benefits of these in the context of multiple deprivation. A limitation of the current study is that it did not seek the views of those in receipt of outreach work and was, therefore, unable to test whether, in the context of an intervention that seeks to address health inequalities, the concept of candidacy needs to be substantially revised.

### Further theoretical and empirical investigation

The current study starts the process of mapping out specific problems underlying non-engagement with preventive health care and of theorising potential pathways between particular problems and health service solutions. It does not, however, track these pathways to determine whether different forms of outreach work in the context of a primary prevention programme impact positively on initial and long-term engagement with the health service or the myriad of interventions provided to ameliorate the social and structural determinants of ill-health. The pathways, once identified, require to be tested through a combination of quantitative and qualitative methods. Longer-term monitoring of service users' changing perspectives on candidacy would be theoretically revealing; as Dixon-Woods and colleagues have argued, the process of accepting and receiving versus resistance to, and rejection of, interventions is under-researched.

Furthermore, work thus far on candidacy has focused on the idea of how candidacy for health services is initiated and negotiated. The inextricable linkages between health and its social determinants, however, warrant the initiation of studies that explore the notion of candidacy within other public and third sector domains.

## Conclusion

This study has demonstrated that non-engagement with preventive health services is a multi-faceted problem and that different strategies are required to tackle its different components. Some of the solutions to the problem are relatively straightforward and within the traditional armoury of primary care, others might be more appropriately tackled using outreach approaches. Outreach approaches, however, have been shown to be heterogeneous and need to be better matched to specific problems and theories of how non-engagement is generated, and tested for their impact on subsequent uptake of services. Dixon-Woods and colleagues' concept of candidacy offers a helpful lens through which to explore non-engagement but needs to more explicitly relate to how material and systems level drivers of service utilisation operate to exclude disadvantaged groups.

## Competing interests

The authors declare that they have no competing interests.

## Authors' contributions

MM, COD, SP and SS were responsible for the design of the national evaluation of Keep Well. MM, COD, SP, FT were responsible for the design of the outreach case-study. Field work and coding was conducted by FT, MR, YW and JC; analysis was conducted by MM, COD, SP, FT, MR, YW and JC. The manuscript was drafted by MM with help from FT. All authors read, contribute to and approved the final manuscript.

## Pre-publication history

The pre-publication history for this paper can be accessed here:

http://www.biomedcentral.com/1472-6963/11/350/prepub
